# A scenario-planning approach to human resources for health: the case of community pharmacists in Portugal

**DOI:** 10.1186/1478-4491-12-58

**Published:** 2014-10-13

**Authors:** João Gregório, Afonso Cavaco, Luís Velez Lapão

**Affiliations:** WHO Collaborating Centre for Health Workforce Policy and Planning, International Public Health and Biostatistics Unit, Instituto de Higiene e Medicina Tropical, Rua da Junqueira n°100, 1349-008 Lisbon, Portugal; Research Institute for Medicines and Pharmaceutical Sciences, Faculdade de Farmácia da Universidade de Lisboa, Av. Professor Gama Pinto, 1649-003 Lisboan, Portugal; Department of Social Pharmacy, School of Pharmacy, The Faculty of Mathematics and Natural Sciences, University of Oslo, Farmasibygningen, Sem Sælands vei 3, 0371 Oslo, Norway

**Keywords:** Community pharmacists, Human resources for health, Pharmaceutical services, Scenario planning, Portugal

## Abstract

**Background:**

Health workforce planning is especially important in a setting of political, social, and economic uncertainty. Portuguese community pharmacists are experiencing such conditions as well as increasing patient empowerment, shortage of primary care physicians, and primary health care reforms. This study aims to design three future scenarios for Portuguese community pharmacists, recognizing the changing environment as an opportunity to develop the role that community pharmacists may play in the Portuguese health system.

**Methods:**

The community pharmacist scenario design followed a three-stage approach. The first stage comprised thinking of relevant questions to be addressed and definition of the scenarios horizon. The second stage comprised two face-to-face, scenario-building workshops, for which 10 experts from practice and academic settings were invited. Academic and professional experience was the main selection criteria. The first workshop was meant for context analysis and design of draft scenarios, while the second was aimed at scenario analysis and validation. The final scenarios were built merging workshops’ information with data collected from scientific literature followed by team consensus. The final stage involved scenario development carried by the authors alone, developing the narratives behind each scenario.

**Results:**

Analysis allowed the identification of critical factors expected to have particular influence in 2020 for Portuguese community pharmacists, leading to two critical uncertainties: the “Legislative environment” and “Ability to innovate and develop services”. Three final scenarios were built, namely “Pharmacy-Mall”, “e-Pharmacist”, and “Reorganize or Die”. These scenarios provide possible trends for market needs, pharmacist workforce numbers, and expected qualifications to be developed by future professionals.

**Conclusions:**

In all scenarios it is clear that the future advance of Portuguese community pharmacists will depend on pharmaceutical services provision beyond medicine dispensing. This innovative professional role will require the acquisition or development of competencies in the fields of management, leadership, marketing, information technologies, teamwork abilities, and behavioural and communication skills. To accomplish a sustainable evolution, legislative changes and adequate financial incentives will be beneficial. The scenario development proves to be valuable as a strategic planning tool, not only for understanding future community pharmacist needs in a complex and uncertain environment, but also for other health care professionals.

## Background

### Community pharmacists’ role in global health systems

Human resources are the present focus of attention in health systems strengthening and public health policies [1–3]. This is partly due to the increase of chronic conditions and the emergence of interprofessional models of practice that aim at transforming the daily care for patients with chronic illnesses from acute and reactive to proactive, planned, and population-based [4]. Most successful chronic illness interventions include major roles for non-physicians such as pharmacists and nurses [5–7]. Community pharmacies and pharmacists are in a privileged position within health care systems due to their professional training and easy accessibility (i.e., in most high streets), which could contribute to more reliable monitoring of medication use and patient counselling, as well as health promotion and education [8, 9].

For pharmacists, this new role towards a more patient-centred care has become a new paradigm of pharmacy practice, leading to the development of patient information services, pharmaceutical care services, and the development of a clinical role for community pharmacists [10, 11]. The work of Hepler and Strand [12], in the early nineties, was a milestone in this change, pointing out to the delivery of longitudinal advanced medication-related services, the rise of professionals’ level of responsibility, and the development of cooperative relationships with other health care professionals as essential features to this new role. However, this movement toward patient-centred care in community pharmacy has been taking longer than one would expect back in the nineties, much influenced by inner organizational barriers as well as several external factors such as the economic and legislative context, commercial pressures, government politics and/or policies, technological innovations, new therapies, support from other professionals, health system integration, and the personal attitudes of pharmacists and pharmacy leaders [10, 11, 13].

### Community pharmacists’ workforce in Portugal

Portuguese community pharmacists have followed the global trend for an extended practice. Community pharmacists in Portugal serve the public in independent shops, the community pharmacies. The installation of community pharmacies is regulated by the government, establishing the minimum distance between pharmacies and number of inhabitants serviced. The number of pharmacies, now close to 2,900 in total, has increased 9.5% since the turn of the century, with an average of 24 new pharmacies per year [14]. From 2007 onwards, changes in legislation allowed for non-pharmacist ownership, a decrease in the population base from 4,000 to 3,500 inhabitants per pharmacy per county, and a shorter distance between pharmacies from 500 m to 350 m. This political measure had impact in that year, with a rapid increase of pharmacies, but quickly stabilized. Pharmacies have a National Health Service (NHS) contract for dispensing medicines, establishing prescription medicines’ profit margins and co-payments. Apart from dispensing, none of the new services is supported by NHS remuneration. To cope with this, pharmacies may offer services such as smoking cessation, minor ailment schemes, and adherence support services, all of which are supported by the patient’s direct payments [15]. During the early 21st century, Portuguese pharmacies have implemented pharmaceutical care programs for hypertension and diabetes with the help of professional organizations such as the National Association of Pharmacies (ANF), a pharmacy owner’s organization. The program for diabetes was financially supported by the NHS from 2006 to 2009. At the time, a maximum of 400 pharmacies were doing patient follow-up, with an average of three patients per pharmacy [16]. After the cancelation of NHS financial support of these programs, most pharmacies terminated the provision of the service and stopped with patient follow-up. Since then, many pharmacies have broadened their services to other services provided by different professionals such as nutritionists, podologists, or nurses in an attempt to have more revenue to face the present financial constraints.

Community pharmacists represent almost two thirds of the total pharmacist workforce mandatorily registered in the Pharmaceutical Society (OF) [17]. By the end of 2012 there were 7,716 registered community pharmacists [18]. These are mostly young professionals (67% less than 45 years old), 80% of which are women. The total number of community pharmacists has increased 74% between 2000 and 2009, with an annual average of 340 newcomers [19, 20]. This sharp rise was a direct consequence of the increase in the number of pharmacy degrees offered in private and public universities. For instance, in 2010 there were more than 1,100 new students enrolled, a 6.5% increase when compared with 2008 admissions [21]. In the same period, the number of pharmacy technicians working in community pharmacy dropped by 25%, mainly due to the oversupply of pharmacists [19, 20]. The ratio of pharmacists per pharmacy has increased between 2000 and 2010, with an average above 2 since 2005, leading to 68 pharmacists per 100,000 inhabitants [20, 22], which can be considered a homogenous geographical distribution, although with a greater concentration in the Lisbon and Porto metropolitan areas [14, 19, 20, 23]. Due to the decrease of pharmacy technicians, pharmacists then started to have increasingly technical tasks to perform, since the development and implementation of new services was not widespread. With the onset of the economic crisis of 2008, low salaries and unemployment, especially among recently graduated pharmacists, started to become a reality [24].

### The need for strategic planning in community pharmacy

The shortage of primary health care (PHC) physicians, the economic and political uncertainty deriving from the economic crisis that started in 2008, and the primary health care reform currently developing in Portugal, are all contributing to a changed climate and have created an opportunity to rethink the role of community pharmacists within the Portuguese health care system.

Strategic planning is essential to assess the efficiency of human resources and health services, since it is an effective tool to address innovative solutions within health systems [25–27]. Although the organizational environment is recognised as an important factor in health care services functioning and development, external environment continuous modifications challenge decision makers and practitioners. For health professionals, this environment could be described by a constant technological evolution, a growing search for patient-focused care, and empowerment of citizens, particularly in terms of health knowledge [28]. For community pharmacists, the changing environment and the shifting in health care demands is pushing them to a continuous adaptation process and a more advanced role in patient care [29].

Due to these uncertainties, which limit the capacity to predict and plan the needed resources accurately, there is now an opportunity to delve more deeply into Portuguese community pharmacists’ future through a thinking exercise supported by the development and analysis of strategic scenarios [30–32]. Undertaking a strategic thinking approach allows for the analysis of different possibilities, without excluding those that seem unlikely. Recent approaches to the issue of pharmacists’ future have focused on workforce supply and demand [33–35], while others have proposed scenarios to depict what the profession could be in the future, from the perspective of interviewed experts [36, 37]. This last case inspired our work since a flexible approach is used and a creative attitude is promoted towards a future vision on the pharmacist profession.

The aim of this study was to develop future scenarios for the community pharmacist profession in Portugal. To achieve this, two main objectives were considered: (i) to analyse the possible evolution of community pharmacists’ role in the Portuguese health care system by building and studying three different scenarios and (ii) to identify the main driving forces and related uncertainties.

## Methods

Strategic scenario analysis does not aim to predict the future. Instead, it aims to construct stories for the future that contribute to the better understanding of the external environment in which an organization is operating in order to support strategic decisions, anticipate difficulties, and assess an organization’s business positioning [38–41]. Scenario development is a validated and useful methodology, where each scenario can be regarded as a “strategic case” or as a “branch of a decision tree” [42]. Its use extends from academic research to more practical issues as business and public administration [31]. Scenarios are the archetypical products of future studies, as they facilitate both the possibility for a deeper and more creative thinking about the future (reducing the risk of being surprised and unprepared) while, simultaneously, enabling the enhancement of the collective awareness (and preparation) over multiple plausible circumstances [43].

Besides an organizational and business strategy, this methodology has also been used in prospective research of academic medical organizations and professional pharmacy-related groups [36, 37, 44]. For this work, it was decided to use the community pharmacists’ perspective. Although pharmacies and pharmacists are closely linked entities, this distinction is essential since the developmental paths of both bodies do not necessarily overlap.

**Figure 1:**
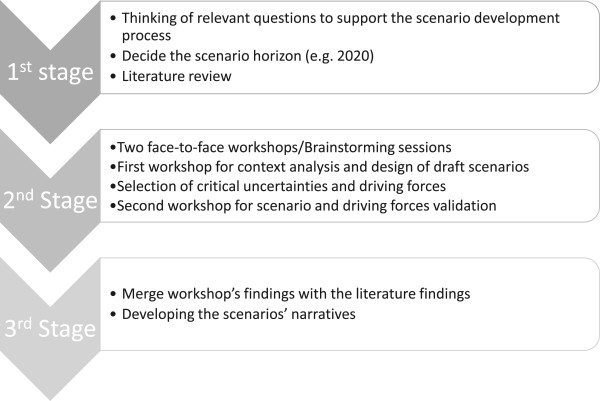
**The three stages of scenario development as proposed by Lapão and Thore** [45] **.**

The method proposed and used by Lapão and Thore was followed [45]. This method condenses the 10-step method of Schoemaker [40] into three stages (Figure 1) that yield a set of three scenarios which represent three different future possibilities. The three scenario development stages are presented in detail below.

### First stage – literature review

In this first stage, the authors thought of relevant questions to support and motivate the scenario development process. For instance, “What will be the need for community pharmacists in the future?”; “Is it possible to establish and sustain a new role for community pharmacists within the health care system?”, “If so, how can community pharmacists integrate the PHC network?”; “What might happen to the newly graduated pharmacists coming to a labour market that is increasingly saturated?” A scenario horizon was also defined. For this study, we selected the 2020 horizon. Ten years is considered a good enough timescale within policy-making, avoiding difficulties in participants’ responsiveness [31].

To perform the literature review, PubMed database and Google Scholar were searched using the following strings: “community pharmacist role”, “community pharmacy future”, “pharmaceutical services”, “pharmacy scenarios”, “health services innovation”, and “pharmacy information technologies”. This search retrieved 167 articles on the global community pharmacists’ role, its evolution, barriers and facilitators to professional development, and future market trends such as the challenges of population ageing or the use of information technologies (IT). The information collected was used to prepare a review to inform the next stage of scenario development. Besides the qualitative information, quantitative information related to Portuguese community pharmacists was obtained from the official “Medicine Statistics” reports, available from the regulatory agency (INFARMED) [19, 20], as well as complementary information from the OF and the ANF [17, 18, 22, 24].

### Second stage – scenario development workshops

The second stage comprised two face-to-face audio-recorded workshops, each with three hours duration. The workshops took place in two different days with the participation of a workgroup that included the first author, a community pharmacy specialist, and a scenario developer. To complete the workgroup, pharmacy and health management experts were invited. A total of 10 experts were invited, 6 of which accepted the invitation (Table 1). These were selected by convenience, using academic experience and professional experience as the main criteria. Academic experience was assessed by relevant publications in the field of community pharmacy. The professional experience was assessed by years of practice and/or ownership of community pharmacy listed on the online CVs. Having community pharmacy experience or health market knowledge in Portugal and being an academic was considered a major asset, although it may be considered a bias toward academia; only one expert was personally known by the researchers. None of the invited experts was an active representative of a professional organization, thus avoiding possible conflicts of interests. Considering previous pharmacist scenarios analysis reported in the literature [36, 37], it was decided that the 8 person group was enough to accomplish our goals.

**Table 1:** **List and characterization of expert informants present at the workshops**

Participant	Sex	Workshop presence		Professional and academic experience
		First	Second	
Expert #1	Male	P	P	Doctor of Pharmacy (PhD), Associate Professor in Social Pharmacy, expert in Pharmacist communication, Community pharmacy co-owner
Expert #2	Female	P	P	Doctor of Pharmacy (PhD), Assistant Professor, expert in Pharmaceutical Care
Expert #3	Male	P	P	Master in Health and Development (MSc), Community Pharmacist specialist; study main author
Expert #4	Male	P	P	Doctor of Systems Engineering and Health informatics (PhD), Assistant Professor, expert in scenario design and trained facilitator
Expert #5	Female	P	P	Doctor of Pharmacy (PhD), Expert in Pharmaceutical Care, Community pharmacy technical director
Expert #6	Male	P	P	Manager, Regional Access Manager for a multinational pharmaceuticals company
Expert #7	Female	–	P	Master in Public Health, Community Pharmacist, expert in Pharmaceutical Care, Community pharmacy co-owner
Expert #8	Female	P	P	Pharmacist, Executive Director of a public primary health care centre cluster

P – Present at the workshop.

The workshops were recorded using a digital recorder with participants’ verbal consent obtained prior to the workshops. The recordings were transcribed and thematic analysis performed. The first workshop was meant for context analysis and design of draft scenarios, while the second workshop aimed at scenario analysis and validation.

According to Godet [38], the first interaction with experts should start with a short seminar to acquaint all participants with the purposed tools and concepts that will be used in the scenario development process. After this introduction, a presentation was made, highlighting the main findings from the literature review, namely on workforce, economic, technological, political, and demographic trends. Next, the process of collecting information started using the thematic brainstorming technique: the experts were asked to imagine different possibilities for community pharmacists’ future role based on whatever their perceptions were on the influence of the literature review findings. This approach allowed a free flow of ideas and discussion, without the boundaries of an interview and conventional reflection. These findings were then summarized into several themes, which is necessary to identify critical uncertainties. Critical uncertainties are environmental factors considered to have an influence in the progression of the theme under analysis [43]. To do so, we asked the participants to vote on the two themes that they thought would be the most influent for the proposed scenario horizon. These themes were then condensed into two major critical uncertainties that will work as the scenarios’ “driving forces”, to develop an initial draft version of the scenarios discussed in the end of the first workshop [46].

To prepare the second workshop, a story for each of the draft scenarios was written, combining the analysis of the workshop’s recording and the selected driving forces. On the second workshop, both the initial draft scenarios and driving forces were checked for consistence and plausibility. This was done by expert consensus. If a scenario or driving force was deemed to be unlikely or if it was incoherent, it would not be considered for further development. Next, the gap between present and future was fulfilled by participants’ discussion aiming to reach a narrative of a consistent set of events that would lead to the three scenarios.

### Third stage – scenario analysis

This third and last stage of the scenario design process was carried by the authors alone. The workshops’ recordings were transcribed and the information collected was combined with information gathered in the literature review to redesign the final set of scenarios, with the corresponding narratives. The narratives are hypothetical stories of the future built by the authors, based on the trends that the invited experts feel that some uncertainties might have on the years to come, their impact on the community pharmacy workforce and on the health system in general.

This study was performed in strict accordance with the good research practices and code of ethics of *Instituto de Higiene e Medicina Tropical, Universidade Nova de Lisboa*, Portugal. The protocol was approved by the Ethics Committee of *Instituto de Higiene e Medicina Tropical*, Universidade Nova de Lisboa (Permit Number: 7-2012- PN).

## Results

During the scenario workshops, several themes considered to be relevant to community pharmacists’ role in the Portuguese health care system were debated. A final voting identified the critical ones. The following descriptions for each of the critical uncertainties were suggested.

### Pharmaceutical services

This was considered the main driver for professional development and professionals’ satisfaction, being also the driver for service differentiation between pharmacies. The experts further considered that there would be more sophisticated services in the future, which will be essential for customer retention. The concept of a “health care mall”, where customers and patients would have access to several health care services provided by different professionals (e.g., nurses, nutritionists, podologists, etc.), was considered highly plausible. When discussing pharmacies’ sustainability, the participants’ belief was that pharmacies would only offer additional services if or when they were profitable.

### Economic environment and financial situation of the pharmacies

The participants agreed with the concept of pharmacies as small enterprises, particularly dependent on NHS co-funding. Presently, pharmacies are facing decreasing profit margins, with new remuneration models that are mostly strangling the smaller pharmacies. The participants’ perceptions about this issue were that pharmacist’s clinical intervention would be in jeopardy as the financial situation of the pharmacies deteriorates, blocking the eventual development of a new role. To counter the decreasing budget trend, pharmacies would be forced to address and improve their management and search for alternative ways of funding. One identified alternative was the clustering of pharmacies, which is already emerging.

### Political will and NHS reorganization

This theme emerged associated with the fundamental idea of a legislative change that would consent new roles to develop. It was recognized that political will is influenced by several aspects, such as the country’s economic and financial situation, pressure from social and economic lobbies, health care professional groups, but also by the relationship between the government party and the main professional organizations, ANF and OF. The awareness of a recent phenomenon – pharmacists’ unemployment – also emerged as a pressure factor for the politicians and professional organizations. Bearing in mind the most significant pharmaceutical policy and economic change in recent years – the loss of pharmacists’ property rights and pharmacies exclusive rights in over-the-counter medicines sales – the participants acknowledged that if the economic situation continues to deteriorate, a change in pharmacy legislation will be inevitable, i.e., there will be pressure to change the minimum distance between providers and adjust the population density limits, allowing for a horizontal and vertical integration of the community pharmacy market. There is also the possibility that the current NHS will suffer liberal reforms, reducing medicine reimbursement and limiting patient purchases.

### Patients and clients

The participants considered that the patients’ perceptions of health care and consequent behaviour influence the demand of products and services. The current economic setting is forcing many patients to choose lower-priced products and fewer services. This effect will influence prices’ policies in order to lower medicine costs even more, through patients’ advocacy groups and other associations. It was considered that the relatively low health literacy of Portuguese patients is an impediment for pharmacy extinction. However, the use of the Internet as an information channel for health issues is growing not only in younger age groups, but also in elders too, a group where chronic diseases are prevalent. This fact, alongside with the perceived early adoption of new information technologies by the Portuguese population, makes Internet based health care and pharmacy services very likely and promising in a near future.

### Professional organizations role

Most workshop participants were certain that the two main professional organizations (ANF and OF) would still be very strong actors in shaping the community pharmacist role, although the ANF will be more dedicated to protect business interests while the OF will maintain the defence of a professional point of view. This will probably happen, assuming that non-owner pharmacists do not necessarily support ANF’s points of view and strategy. The ANF has been advocating the implementation of a new practice model since the late nineties with the OF also supporting this change. However, ANF’s economic power and political influence still places it as a main stakeholder in this arena, with participants recognizing that in the future, it would be better for the profession to keep the OF as the main stakeholder to clearly separate the business interests of pharmacies from the professional interests of non-owner pharmacists. Besides this, the role of academia was also deeply discussed. It was recognized that academia will have an important role in defining pharmacist education and thus, their specialization toward a patient centred practice.

### Primary health care reform

The present reform is promoting the harmonization of clinical procedures between primary and secondary health care, and this will also influence pharmacies’ organization, management, and positioning. The integration of a pharmacist in PHC centres’ teams emerged as a possibility. Depending on the functions performed (e.g., disease management versus medicine use management and logistic support) there could be a stronger link with the local community pharmacies to better integrate the patients in the health care network. To make this possible, participants suggested pharmacists should acquire new communication skills to better work with other health professionals, and taking into account factors such as lobbying, trust between professionals, and cultural issues.

### Other themes

From the several themes discussed that were not considered critical uncertainties, it is worth to highlight themes such as “absence of a community pharmacist career”, implying that the professional development is non-existent and the specialization occurs mainly within the practice setting; technological innovations will surely play a major part in shaping the future pharmacist’s role, but could have their potential hindered by most “patients’ low IT literacy”; the “inconsistency of services between pharmacies” was identified as one barrier to the dissemination of new forms of practice, contributing to a low speed of diffusion of innovations.

### Scenarios’ driving-forces

To start the scenario design process it was necessary to condense the critical uncertainties into two driving forces. The first driving force was named “Legislative environment”, comprising the critical uncertainties “Political will and NHS reorganization”, and “Economic environment”. Two extremes for this driving force were considered, namely one with little or no change in the legislative and economic environment, and the other comprising both pharmacy market and health care system liberalization.

The second driving force was named “Ability to develop services”, condensing the critical uncertainties “Financial situation of the pharmacies” and “Patients and clients”. The extremes for this driving force were low innovation versus high innovation that enables new service development. The first was due to the absence of incentives, lack of demand, and lack of pharmacies’ financial capacity, resulting in a low development of new services. The second emerged from greater customer demand, resulting in an increased need for differentiation between pharmacies as a means to expand their client base and profits.

**Figure 2:**
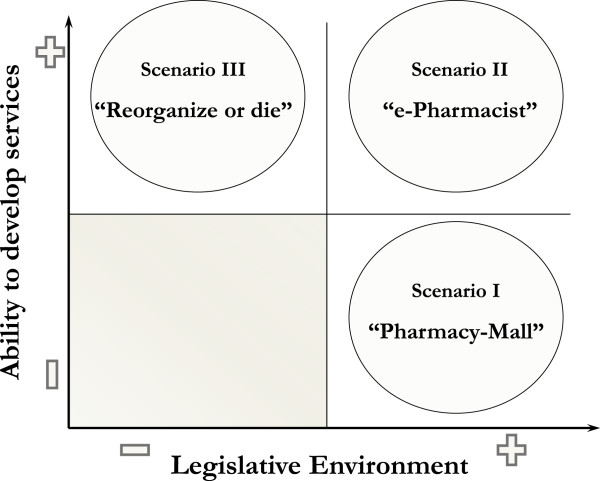
**Final community Pharmacists’ scenarios.**

After the driving forces were fully defined and validated by the experts, it was possible to design the following final scenarios (Figure 2).

### “Pharmacy-Mall”

In the “Pharmacy-Mall” scenario, new pharmacies’ opening and ownership will have been completely liberalized, but with no significant modification in the way pharmacies are paid for their services. Remuneration will come entirely from the profit margins on the sale of medicinal products. There will be no governmental attempts to improve services’ development or payment for the existing ones. As the profit margins improve, due to the horizontal and vertical integration of pharmacies into chains, pharmacies’ owners will not feel the need to innovate, thus keeping a low development of new pharmaceutical services. The demand for pharmaceutical services will also remain low. There may be pharmacies in the Internet, providing medicines to patients who cannot or do not want to visit the pharmacy to fill their prescriptions.

### “e-Pharmacist”

In the “e-Pharmacist” scenario, besides pharmacies’ ownership liberalization, there is the possibility of health system liberalization, i.e., greater opportunities for health services’ personalization. In this sense, it will be beneficial for the citizens to have a health insurance or a subsystem of care. In this scenario, pharmaceutical care services become the main source of revenue for pharmacies. Pharmacies will be mainly supported by private health systems, thus encouraging the development of additional pharmaceutical services. Community pharmacists, skilled in the provision of services beyond medicine dispensing, will be subject to a strong demand. They will be recognised for the economic and clinical value in the services provided and the impact on patients’ quality of life will be definitively proven. Service innovation will lead to a broader utilization of IT: proactive community pharmacists will be showing leadership on the use of information systems for provision of health care, managing virtual spaces, such as electronic cabinets, where all the disease and therapeutic management will be accomplished. “e-Pharmacists” will manage patient’s health information and the interaction with PHC physicians and other health professionals using digital and IT resources.

### “Reorganize or Die”

In the “Reorganize or Die” scenario, no significant legislative changes will have occurred, other than cutting profit margins. The current trend for decreasing profit margins will continue, forcing pharmacies to look for other sources of revenue, including reorganizing in groups of pharmacies. This will help survival and will maintain the pharmacies’ minimum profit level and costs controlled. The development of services will take place based upon the need to differentiate between pharmacies, implementing strategies to increase their products and services’ demand. In this scenario, pharmaceutical services would only exist if supported by individual payers, without any financial support from the health systems. Most pharmacies will have their own Internet site; however, since there will be no legislative change, dispensing prescription medicines will still be performed in the traditional way. The websites will be more dedicated to the sale of over-the-counter products and provision of general health information.

## Discussion

From the analysis of our set of scenarios, it seems that the most promising future for community pharmacists in Portugal is the provision of pharmaceutical services that go beyond medicine dispensing.

Comparing this set of scenarios with others found in the literature, namely the scenarios suggested by Norgaard et al. [37] for Danish community pharmacists, we can conclude that much of the trends that led to the development of their scenarios are still very much present today, leading us to believe that the transition in pharmacist role is universal but has been much slower than expected. In that work, five scenarios were developed using a different methodology. Apart from the “No future scenario”, which is a “worst case scenario”, that we did not consider developing, there are several common aspects with the remaining scenarios. Their “Uncertain future” scenario is closely related to our “Pharmacy-Mall”. However, aspects of their “IT expert” and “Provider of individualised information and future role developer” scenarios are included in the “e-Pharmacist” scenario, with some other aspects being included in the “Reorganize or Die”. This may stem from different methods approaching the scenario development, as the perception of pharmacist’s role is very similar in both countries. Although different in their context, these scenarios show some intriguing trends in spite of the “Danish” scenarios lack of a clear scenario horizon. The trend towards the use of technological solutions to assist pharmacist’s work is present as is the fear of becoming less important in the health system.

The choice of the two driving forces from the themes discussed was a critical step in the definition of our scenarios. They were discussed and validated by the experts at the beginning of the second workshop. For the first driving force, “Legislative environment”, the decision was supported by the perception that the most important change in the pharmacy sector in Portugal, and also in Europe, is the liberalization of pharmacies’ ownership and installation [23, 47, 48]. Although some authors found that restrictions to free pharmacy installation are limiting innovation [23], other studies suggested that the current legislation on pharmacy installation ensures equity of access and the quality of medicine dispensing as long as the presence of a pharmacist is mandatory [47–49]. Experts’ recognition that these contrasting views stem from the political environment, supported the integration in this driving force of the issues of “political will” and “economic environment”. The second driving force “Ability to develop services”, intends to reflect the competency, will and vision of a pharmacy owner to develop and implement innovative services. The experts considered that the development and implementation of pharmaceutical services is critical for pharmacy differentiation and sustainability, which in turn impacts on the community pharmacist role. It also acknowledges that pharmacy owners will only have interest in implementing services that will be profitable, with adequate service remuneration as an essential requirement for ensuring service diffusion and adoption, as described in the literature [13, 48]. It is expected that pharmacy owners will adopt this new practice, especially in a context of economic constraints. However, the lack of business skills and openness to innovation could represent a barrier to this process of change.

The final three scenarios represent obvious implications and consequences, which are summed up in Table 2.

**Table 2:** **Implications and consequences of the different scenarios**

	Scenario I	Scenario II	Scenario III
	“Pharmacy-Mall”	“e-Pharmacist”	“Reorganize or Die”
**Demand for community pharmacists**	• Decreases	• Increases	• Decreases
**Main functions and responsibilities**	• Supervision of dispensing processes	• Caregiver	• Innovator/salesman
**Main skills to acquire/improve**	• Leadership	• Clinical pharmacy and pharmacotherapy	• Client management
	• Human resources and pharmacy management skills		• Marketing skills
		• Advanced information technologies	• Sales techniques
	• Pharmacovigilance		• Communication skills
	• Information technologies	• Teamwork abilities	
	• Regulatory/reimbursement affairs		
**Primary health care integration**	• No integration	• Multidisciplinary teams and polyclinics	• In the local health units

**Figure 3:**
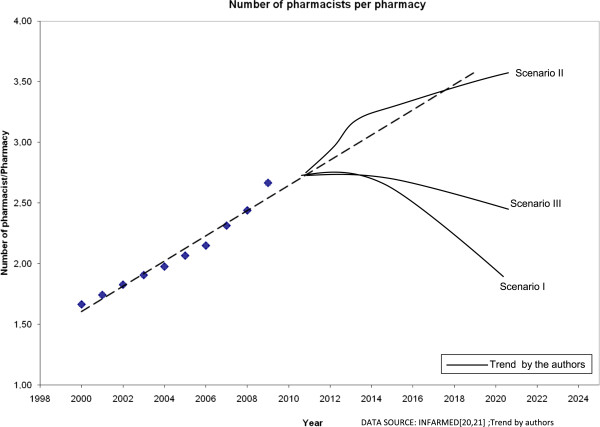
**Evolution of the ratio of pharmacists per pharmacy in each scenario.**

The “Pharmacy-Mall” scenario is the one that offers community pharmacists fewer chances for professional development, besides representing a likely sharp decrease in workforce numbers (Figure 3). The potential oversupply of pharmacists and rising unemployment will contribute to the increasing number of professionals leaving the country, seeking new job opportunities, professional development, economic stability, and job satisfaction [50]. Others will quit the profession, choosing another career outside community pharmacy [51]. In a scenario where big profit-driven pharmacy chains will emerge, conflicts are expected between business objectives and pharmacists’ interventions, namely “free of charge” patient counselling. This can have negative consequences on professional satisfaction and community pharmacists’ work conditions [52, 53]. For patients, the advantage of this scenario is the reduction of medicine prices, due to greater vertical and horizontal integration and additional competition [47]. For the health system, this scenario could bring important savings in medicine expenditures, as it has been observed in countries with a similar model. Nevertheless, the downside is a reduced accessibility to medicines, especially in remote and rural areas [47, 48].

The “e-Pharmacist” scenario represents a “best case scenario”. The numbers of community pharmacists per pharmacy will keep rising, ensuring that a significant part of the trained pharmacists is still absorbed by the community pharmacies market (Figure 3). However, it is crucial for pharmacy professionals to promote a greater collaborative work culture, both inside and outside the pharmacy. This scenario implies a greater collaboration with general practitioners and nurses, and probably some changes in working regulations. With more information to share, the use of IT is an evident solution that will continue to be explored [54, 55]. For patients, this may be a valuable scenario since there is the possibility of remote disease and therapy management, associated with better health outcomes, less general practitioner visits and other health care system savings [6, 56, 57]. It is likely that an improved accessibility will increase patient satisfaction. For the health care system, a real integration of community pharmacists in the PHC network would possibly increase the costs of care. However, the efficiency increment and the reduction of other costs would be relevant to those financing the health system, without affecting the quality of pharmaceutical services provided [58, 59].

In the “Reorganize or Die” scenario, customer relations management competencies are the most valuable asset a pharmacist can have. A solid education in marketing and quality of services will be extremely important to increase demand for the products and services each pharmacy is willing to provide. For patients, this scenario is not as valuable as others, since they will be considered as consumers rather than actual patients using a health service. In this sense, not everyone will have access to pharmaceutical care services, depending on the services available at each pharmacy and on the individual means to pay for services. Since funding of pharmaceutical services will be largely dependent on patients, the overall equity of the health care system could be endangered, since most of the people who might need pharmaceutical care may not be able to afford it. As far as the health care system is concerned, besides the lower equity, the absence of community pharmacists’ integration in the PHC network will limit the gains in efficiency associated to pharmaceutical care [60].

### From scenarios’ to reality – roadmap to develop pharmacist’s new role

One can argue that to sustain the benefits of the envisioned change, an integrated and longitudinal perspective of services’ provision should be considered, requiring event registering, comprehensive data analysis, and interactive dialogue with patients, i.e., enabling a more sophisticated use of information systems. Health care services should be based on health knowledge, people, and technology supporting health care processes. Future pharmaceutical care services should be developed to first deliver valued information to patients and health professionals. This could only work if community pharmacists assume their role as caregivers, supported by adequate information systems, developing caring abilities, and also taking responsibility for patients’ therapeutic results, which is essential to the practice of pharmaceutical care and other professional services [61]. However, this patient-centred practice will also require curricula adaptation towards clinical practice [62–64], without losing sight of all issues related to drug discovery, development, delivery, and use, from applied pharmacology to pharmacoepidemiology.

Information systems will have an important impact on the definition of new roles for community pharmacists [10, 65–68]. The proper use of technological solutions could relief pharmacists’ workload, sparing time to perform pharmaceutical care functions. These functions will be supported by new technological solutions such as the remote monitoring of patients [56, 69, 70].

The relation with other health professionals is also essential to the diffusion of this new kind of practice and for the real integration of community pharmacists in primary health care. Firstly, the role of pharmacy technicians has to be clarified. Nowadays, pharmacists are the larger workforce in Portuguese community pharmacy, being required to perform activities that could be performed by technicians with more efficiency. In an exploratory study made recently, Lapão et al. [71] found that pharmacists and technicians are doing the same activities, with pharmacists spending 40% of their time on non-professional tasks. From these findings, it is clear that a better organization of internal functioning of pharmacies is needed, with precise role definition, delegation of tasks, and supervision mechanisms in place if Portuguese community pharmacists want to move beyond medicine dispensing.

A good interprofessional relationship with physicians is an important factor in integrating pharmacists in the PHC network as are interpersonal skills and an adequate communication with all elements of the health care team [72–74]. However, the relationship with physicians is frequently reported as a major barrier to the development of pharmacists’ new roles in the community [75, 76]. Moreover, nurses have taken roles in primary care provision that could be performed by pharmacists, and this evolution within the health care team is something that community pharmacists should take into account [77, 78]. It is important for the community pharmacists to be aware of other health professionals’ competencies and skills and vice-versa, probably through educational sharing at the university level, in order to stimulate synergisms which best serve the community health needs.

### Future research

This kind of study is particularly important to generate new research questions that will help design the best strategies to enforce an effective change in Portuguese community pharmacists’ role. Below are some examples of possible questions that followed the scenario analysis.

### Emerging research questions

-  What will the impact of the International Monetary Fund/European Central Bank Memorandum of Understanding in the pharmacy business be;
-  What impact will the reorganization of the community pharmacy sector in the role of the community pharmacist have;
-  What will the future needs of community pharmacists in Portugal be;
-  What expectations do the community pharmacists have for their future;
-  What services will patients really need from community pharmacists;
-  Are today’s community pharmacists curricula adapted to future practices;
-  What would the impact of a community pharmacist working in PHC, either in a health care centre or in a health care trust, be;
-  What new information technologies may be used, how should these be used, and what impact will they have in developing new services.

Further, it will be interesting to define a set of indicators that enables the monitoring of community pharmacists’ role evolution. The information gathered in this indicators would help to support better policy making and human resources planning.

### Limitations

The scenarios here depicted do not intend to predict or in any way define the future of Portuguese community pharmacists. Rather, they should be seen as a way to frame possible futures, in order to stimulate new forms of practice and prepare the best strategies in an ever-changing society [41]. Bearing this in mind, the choice of two driving forces and the use of a 2 × 2 matrix may result in a set of arbitrary scenarios. If other driving forces were chosen, different scenarios would be designed. With the methodology described herein we intended to choose the driving forces that seemed to better frame all possible futures, considering them as a starting point instead of a fully developed design.

This methodology is somewhat subjective, which results in a process easily weakened by some “traps” that are usually related either to the way this process is conducted inside an organization (team composition; brainstorming vs. interviews) or to the scenarios’ time frame (short-term vs. long-term). One of the most common “traps” that planners face when developing scenarios is to consider the scenarios as a fixed prediction of the future or to bet in only one scenario, instead of looking at alternatives. The scenario analysis is a flexible process meant to be adjusted to future developments. All these difficulties were considered as a natural part of a scenario planning such as this, but they can also be seen as a weak point of this methodology.

## Conclusions

The use of scenario analysis in a strategic thinking process has demonstrated to be of value while planning for future human resources and other policy issues. It creates a good setting for stakeholders to be more involved, and discuss and study common issues. With the present scenarios, it is possible to anticipate future community pharmacists’ needs, at market and educational level, thus providing valued information to health regulators and planners.

From these scenarios, it is clear that the foreseen changes in pharmacy practice will potentiate the development of new roles for Portuguese community pharmacists in the future health care system. The new role will require significant legislative changes, adequate financial incentives and other behavioural changes, namely an entrepreneur mind-set and innovator’s dynamics. Ideally, the new role will balance the traditional dispensing with a clinical orientation, emerging from pharmaceutical care practice and disease managing programs. Defining a model to finance these services will be vital to preserve community pharmacists’ contribution and the overall equity of the Portuguese health care system.

Changing all parties’ perceptions, from patients to other health professionals, health authorities, and community pharmacists, is critical for embracing a new paradigm in pharmaceutical services provision. In this collaboration, properly designed information systems and technologies will have a very important role, opening the opportunity for community pharmacists to assume true responsibility for patient and disease management.

The practice change will imply new ways of working and interacting with patients, physicians, and other PHC professionals. The envisaged practice change, proposed by the prospective scenarios, would only be effective if all involved professionals are included. The professional organizations have now to show leadership and coordinate strategies to ensure that the new practice might reach all practitioners in a near future for the benefit of the health care system.

## Abbreviations

ANF: National Association of Pharmacies
IT: information technologies
NHS: National Health Service
OF: Pharmaceutical Society
PHC: primary health care.
